# Quantum Dots for Tracking Dendritic Cells and Priming an Immune Response *In Vitro* and *In Vivo*

**DOI:** 10.1371/journal.pone.0003290

**Published:** 2008-09-29

**Authors:** Debasish Sen, Thomas J. Deerinck, Mark H. Ellisman, Ian Parker, Michael D. Cahalan

**Affiliations:** 1 Department of Physiology and Biophysics, University of California Irvine, Irvine, California, United States of America; 2 Department of Neurobiology and Behavior, University of California Irvine, Irvine, California, United States of America; 3 National Center for Microscopy and Imaging Research, Center for Research in Biological Structure and the Department of Neurosciences, University of California San Diego, La Jolla, California, United States of America; University Paris Sud, France

## Abstract

Dendritic cells (DCs) play a key role in initiating adaptive immune response by presenting antigen to T cells in lymphoid organs. Here, we investigate the potential of quantum dots (QDs) as fluorescent nanoparticles for *in vitro* and *in vivo* imaging of DCs, and as a particle-based antigen-delivery system to enhance DC-mediated immune responses. We used confocal, two-photon, and electron microscopies to visualize QD uptake into DCs and compared CD69 expression, T cell proliferation, and IFN-γ production by DO11.10 and OT-II T cells *in vivo* in response to free antigen or antigen-conjugated to QDs. CD11c^+^ DCs avidly and preferentially endocytosed QDs, initially into small vesicles near the plasma membrane by an actin-dependent mechanism. Within 10 min DCs contained vesicles of varying size, motion, and brightness distributed throughout the cytoplasm. At later times, endocytosed QDs were compartmentalized inside lysosomes. LPS-induced maturation of DCs reduced the rate of endocytosis and the proportion of cells taking up QDs. Following subcutaneous injection of QDs in an adjuvant depot, DCs that had endocytosed QDs were visualized up to 400 µm deep within draining lymph nodes. When antigen-conjugated QDs were used, T cells formed stable clusters in contact with DCs. Antigen-conjugated QDs induced CD69 expression, T cell proliferation, and IFN-γ production *in vivo* with greater efficiency than equivalent amounts of free antigen. These results establish QDs as a versatile platform for immunoimaging of dendritic cells and as an efficient nanoparticle-based antigen delivery system for priming an immune response.

## Introduction

As professional antigen-presenting cells of the immune-system, DCs are instrumental in the initiation of adaptive immunity by presenting antigen to T cells [1]. Immature DCs endocytose soluble molecules and particulate material from the surrounding medium via macropinocytosis and adsorptive endocytosis, and then migrate from peripheral tissues to draining lymph nodes [2]. Antigenic proteins are processed into peptides, and loaded to major histocompatibility (MHC) molecules class I and class II. In immature DCs, MHC-II is present in late endosomes or lysosomes [2]–[5]. However, with time, MHC-II molecules are trafficked via class-II vesicles towards the plasma membrane, where the pMHC-II is presented to T cells [4]. DC maturation, characterized by reduced endocytosis and by upregulation of the cell-surface activation markers CD80 and CD86, is promoted by “danger” signals or ligands for toll-like receptors [6]. Although the chemistry and subcellular distribution of compartments related to the antigen-presentation pathway in DCs have been studied extensively, the real-time dynamics of early endocytic vesicles in DCs have not yet been characterized.

Previous investigations have established two-photon microscopy as an elegant method for imaging deep inside a living tissue or organ for long periods (hours) [7]. The spatial and temporal resolution achieved by two-photon microscopy is unparalleled by other methods of non-optical deep-tissue imaging, including PET and FMRI [8]. Previous imaging studies in the lymph node utilizing fluorescent ‘cell-tracker’ dyes have visualized skin-draining DCs carrying cognate antigen, and the associated dynamic changes in T cell motility induced by DC contact [9]–[12]. Initially, T cells make transient contacts (stochastic scanning) with antigen-presenting DCs, and their behavior then progresses from serial interactions to formation of dynamic T cell clusters, T cell dissociation and swarming, leading ultimately to several rounds of T cell proliferation.

Quantum dots (QDs) are semiconductor nanocrystals that exhibit very bright and photostable fluorescence. QDs have broad absorption spectra, and narrow Gaussian emission spectra with peak emission wavelengths that depend upon their size [13], [14]. They are available encapsulated with biopolymeric coatings that allow conjugation with biomolecular moieties, and have been used for imaging in several *in vitro* and *in vivo* biological systems [13], [15]–[17]. When observed by two-photon microscopy QD fluorescence is 100–1,000 times brighter than conventional fluorophores, and is maintained for long periods of time without photobleaching. Proteins can be stably conjugated to the surface of encapsulated QDs by charge-charge interaction, or by using linkers such as streptavidin and maltose-binding protein. In addition to fluorescence imaging, individual QDs can be observed using electron microscopy, and correlated with fluorescence microscopy [18]–[20]. Although QDs have been used for high-resolution imaging in biological systems, they have not yet been used for simultaneous modulation of biological function.

We have harnessed the fluorescence and bioconjugation potential of QDs for real-time imaging of dendritic cells (DCs) *in vitro*, for deep tissue imaging of DCs *in vivo*, and for concomitant modulation of DCs to elicit an immune response to a specific antigen both *in vitro* and *in vivo*. The broad excitation spectra and narrow emission spectra of QDs make them ideally suited for two-photon fluorescence excitation and color separation from other probes. In addition, the high two-photon cross-section of QDs greatly increases their signal to noise ratio as compared to fluorescent proteins. These two properties facilitate detection of QD labeled cells by two-photon microscopy during real-time tissue imaging. Moreover, the ability to conjugate QDs with bioactive proteins makes them potentially useful for modulating biological responses and for cell tracking. Here, we first illustrate the use of QDs for *in vitro* imaging of intracellular compartments inside DCs following endocytosis. We then demonstrate that QDs facilitate real-time tissue imaging of DCs inside the lymph node. Finally, we show that quantum dots can be used effectively as a particle-based antigen delivery system, employing specific antigenic proteins conjugated to QDs to trigger T cell activation with high efficiency in two transgenic TCR models.

## Results

### Quantum dot uptake by dendritic cells

**Figure 1:**
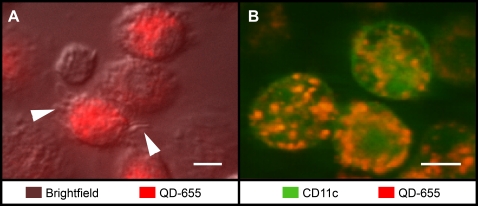
DCs avidly endocytose QDs. (A) Overlay of brightfield and fluorescent images showing endocytosed QD-655 (red) within DCs with dendritic processes (indicated by arrows), after 30 min of incubation of DCs in QD-containing medium. Image is a single frame from Video S1. Scale bar = 10 µm. (B) Two-photon image showing endocytosed QDs (red) inside CD11c^+^ DCs (green) cultured from bone marrow of transgenic EYFP-CD11c reporter mice. Scale bar = 5 µm.

Murine DCs derived from bone marrow avidly endocytosed streptavidin-conjugated or unconjugated QDs during imaging *in vitro* at 37°C. QDs differing in size from 15–20 nm were taken up at approximately the same rate (not shown), and were retained for at least 48 hr without toxicity (Figure S1). In these mixed cultures, cells that became brightly labeled by taking up QDs also exhibited dynamic dendritic processes (Figure 1A; Video S1), whereas the smaller, round cells (presumptive monocytes) did not take up QDs. Flow cytometric analysis showed that QD^+^ cells also expressed the characteristic DC marker CD11c (Figure S2). Furthermore, in experiments on bone marrow-derived cells cultured from EYFP-CD11c reporter mice, EYFP^+^ DCs became brightly labeled with endocytosed QDs (Figure 1B). Together, these results show that QDs are avidly and preferentially taken up by CD11c^+^ DCs.

**Figure 2:**
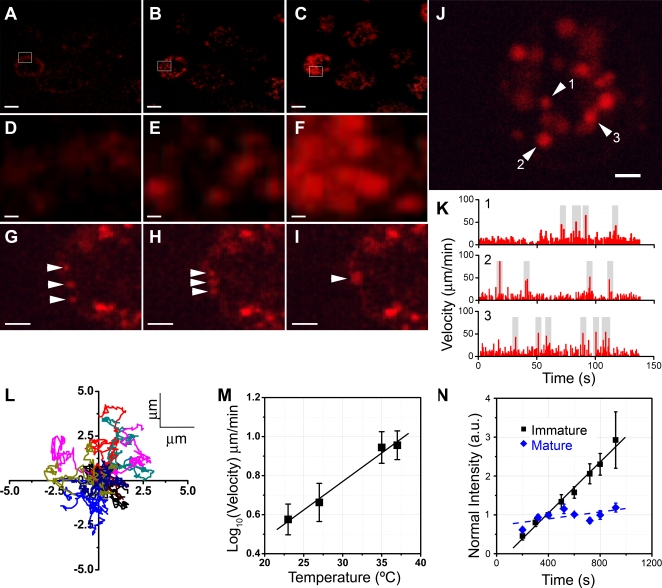
Dynamics of QD-uptake and QD-containing vesicles. (A–F) Two-photon images of QD uptake by DCs at ∼2 min, 9 min, and 18 min after beginning incubation with 2 nM QD-containing medium at 37°C. These frames have been taken from from Video S2. Indicated areas of A–C are magnified in D–F respectively to show individual vesicles. Scale bars = 10 µm in A–C, and = 1 µm in D–F. (G–I) Fusion of QD-containing vesicles inside DCs. Three vesicles, indicated by arrows, come closer, and eventually fuse into a single larger vesicle. These frames have been taken from Video S3. Scale bars = 2 µm. (J) DCs with QD-containing. The image is a single frame taken from Video S4. Scale bar = 4 µm. Arrowheads with numbers represent three individual vesicles, whose velocities are shown in K. (K) Velocity profiles of the individual vesicles indicated by numbers in J. Velocity bursts (with peak velocities >5 times the average velocity) are marked in gray. (L) Trajectories of 10 individual vesicles that could be tracked for >140 sec, normalized to their starting coordinates. (M) Temperature-dependence of vesicular velocities in DCs. Data from 3 independent experiments, 10–12 vesicles per experiment. (N) Downregulation of rate of QD-uptake by DC maturation. Plot of normalized mean QD-fluorescence intensity over time for LPS-treated or mature (diamonds) and untreated or immature (squares) DCs. Lines are linear regressions, with slopes of 32.8×10^−4^ a.u./s for immature cells and 4.4×10^−4^ a.u./s for mature cells. Data from 4 independent experiments for each condition, 6–10 cells per experiment.

We monitored the kinetics of QD uptake following addition of QDs to the bathing medium (Figure 2A–2F; Video S2). QD-fluorescence appeared within 2 min as a band adjacent to the plasma membrane of DCs (Figure 2A and 2D). Subsequently, distinct QD-containing vesicles were apparent inside the cells, initially close to the plasma membrane (Figure 2B and 2E), and later throughout the cytoplasm (Figure 2C and 2F). At longer times, the number of QD-containing vesicles per cell increased, as did their size and fluorescence intensity. Close inspection of single cells showed individual vesicles of varying size (up to ∼1 µm in diameter) ‘jiggling’ in the cytoplasm. Occasionally, vesicles fused with one another inside the cell (Figure 2G–2I; Video S3), but vesicle fusion with the plasma membrane or release of their contents was never observed. Individual vesicles moved in a “stop and go” manner, exhibiting occasional bursts of high velocity followed by periods of jiggling (Figure 2J and 2K; Video S4). The mean vesicular velocity was 9±1.5 µm/min at 37°C, whereas peak velocities during bursts were 5–10 times faster. Vesicular velocities were independent of the apparent variation in vesicular sizes. Vesicles did not exhibit any directional preference in their motion, as shown by their trajectories (Figure 2L). Increased temperature reversibly increased vesicular velocities (Figure 2M; Video S4) with a Q_10_ of ∼2. The rate of endocytosis is known to slow following maturation of DCs [2]; and, consistent with this, we observed a dramatically (10-fold) reduced rate of quantum dot uptake in DCs matured by treatment with LPS (Figure 2N). QD uptake was observed in a greater proportion of cells in the immature-DC population compared to matured DCs (Data not shown). The number of QD-containing vesicles was reduced in LPS-treated DCs. However, velocities and subcellular kinetics of vesicles was not altered.

**Figure 3:**
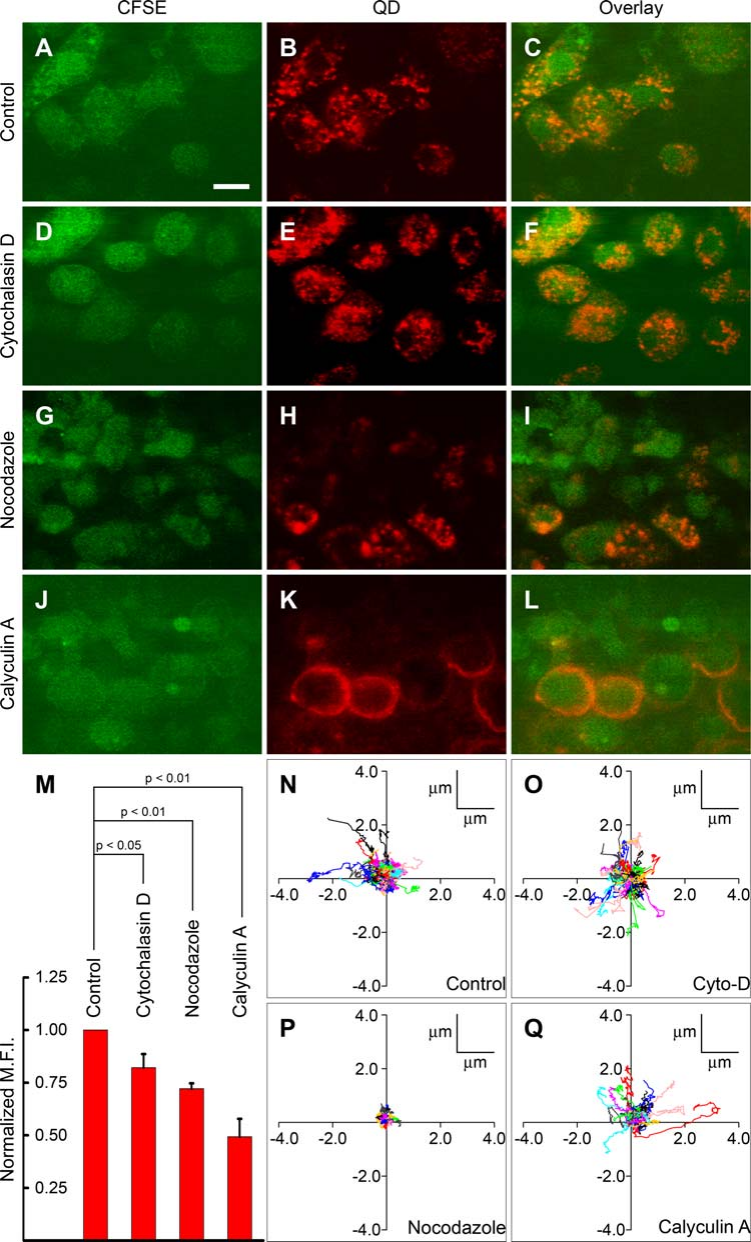
Effects of cytoskeletal inhibitors on QD-endocytosis by DCs. QD (red) uptake in untreated control DCs (A–C), or DCs treated with cytochalasin D (D–F), nocodazole (G–I), or calyculin A (J–L). Scale bar = 10 µm. (M) Histogram showing mean normalized QD-fluorescence intensity of cells treated under different conditions as indicated. QD-fluorescence intensity has been expressed as a ratio of the mean QD-fluorescence intensity of untreated DCs. (N–Q) Trajectories of QD-containing vesicles inside untreated control DCs (N), or acutely treated with 1 µM cytochalasin D (O), 1 µM nocodazole (P), or 200 nM calyculin A (Q), respectively. The effects of these reagents on the dynamics of QD-containing vesicles inside DCs is further illustrated in Video S5, S6 and S7.

To investigate the involvement of cytoskeletal elements on QD uptake and vesicle dynamics, we observed effects of pre-treatment and acute addition of cytochalasin D, calyculin A, or nocodazole in DCs (Figure 3). Untreated control DCs displayed QD-containing vesicles, as well as intact actin filaments (Figures 3A–3C and S3A–S3C). Actin filaments were absent inside cytochalasin D-treated DCs, whereas patches of condensed actin were observed near the plasma membrane along with vesicles that contained QD (Figure S3D–S3F), and total uptake of QD was partially inhibited (Figure 3D–3F, 3M). Inside nocodazole-treated DCs actin filaments were intact and QD-containing vesicles were apparent, as in untreated DCs (Figures 3G–3I and S3G–S3I). Nocodazole-treated DCs rounded up and total QD uptake in these DCs was again partially reduced compared to untreated DCs (Figure 3M). Calyculin A-treated DCs rounded up, and cortical actin was observed condensed near the periphery (Figure S3J). QD-uptake was abolished in most calyculin A-treated DCs (Figure 3J–3M, S3J–S3L). When present, QD fluorescence was restricted to the cell perimeter. However, deeper intracellular QD-containing vesicles were not formed. Together, these results indicate that the presence of free cortical actin is necessary for QD-uptake by DCs.

We next studied the effects of these cytoskeletal inhibitors on the dynamics of QD-containing vesicles within DCs. Cytochalasin D did not affect vesicular dynamics (Figure 3O, Video S5). However, nocodazole produced vesicle arrest (Figure 3P, Video S6), implying that microtubular assembly is necessary for motion of individual vesicles inside DCs. Moreover, in untreated DCs, vesicles did not freely diffuse inside the cytoplasm, but exhibited constrained “jiggling” movement (Video S4), consistent with an attachment to microtubules. Calyculin A did not inhibit vesicular motion. (Figure 3Q, Video S7). In calyculin A-treated DCs, vesicles were occasionally observed to traverse longer paths compared to untreated DCs, indicating the absence of interactions with actin for relatively longer periods of time.

### Compartmentalization of endocytosed QDs

**Figure 4:**
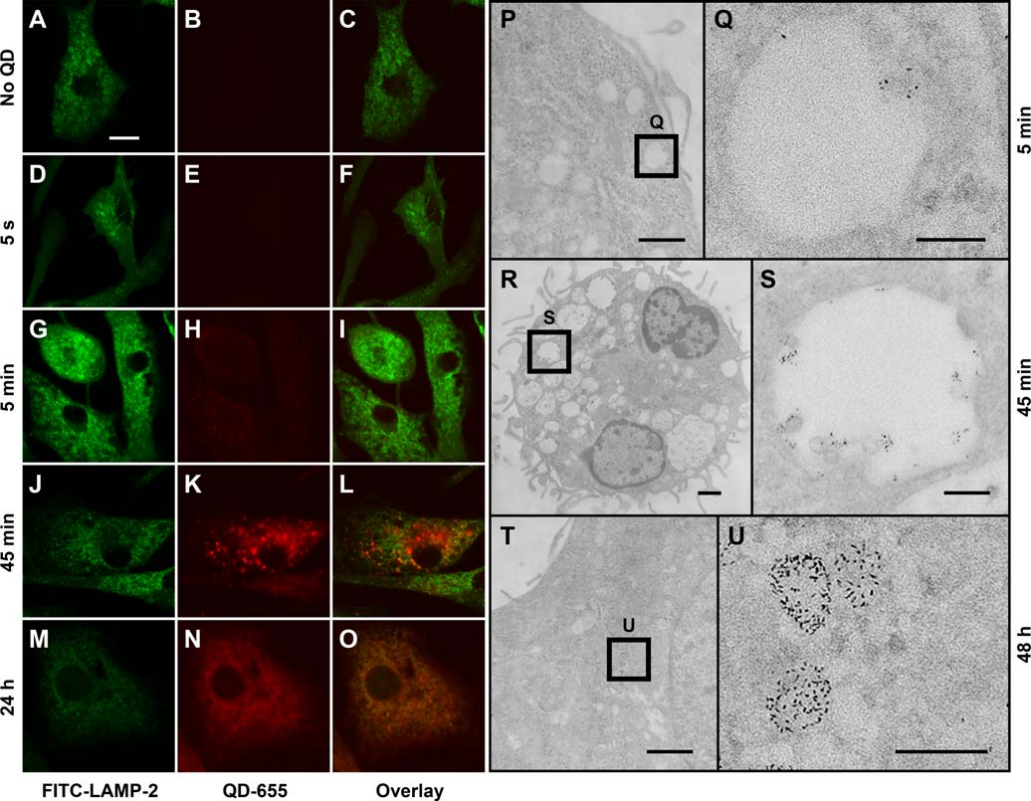
Compartmentation of single QDs inside endocytic vesicles and lysosomes. (A–O) Confocal images showing colocalization of QD-containing vesicles with the lysosomal protein LAMP-2 inside bone marrow-derived DCs after different times of incubation in QD-containing medium as indicated on the left. Columns show LAMP-2 staining (green), QD staining (red), and overlays of LAMP-2 and QD images, respectively. Scale bar in A–O = 10 µm. (P–U) Electron mircographs showing individual QDs within DCs, and associated structures and location after different times of incubation in QD-containing media as indicated on the right.. Indicated regions in P, R, and T, respectively, shown at higher magnification. Note the correspondence between electron micrographs P, R, and T with confocal images I, L and O, respectively. Scale bars in P, R, and T = 1 µm and in Q, S, and U = 200 nm.

Co-labeling of QD-containing DCs for the lysosomal protein LAMP-2 showed that QD-containing vesicles (within 0–5 min of incubation) did not colocalize with lysosomes initially (Figure 4A–4I). However, at later time points (>∼45 min) an increasing number of QD-containing vesicles colocalized with lysosomes (Figure 4J–4O), indicating that eventually QDs are retained inside lysosomes. We also visualized the distribution of single QDs inside DCs by electron microscopy (Figure 4P–4U). QD-containing vesicles were localized near the plasma membrane after 5 min of incubation (Figure 4P and 4Q); at this time vesicles typically contained ∼5–6 QDs and had sizes <500 nm. After 45 min of incubation the numbers of QD-containing vesicles increased, each containing ∼20–30 QDs, with sizes up to 1000 nm (Figure 4R and 4S). Vesicles of varying size were distributed throughout the cytoplasm. As expected, QDs were excluded from the nuclei and mitochondria. Together, light microscopy and em visualization indicate that DCs continually endocytose QDs into small vesicles that subsequently fuse to form larger vesicles that at later times mature into lysosomes with higher electron density containing ∼50–100 QDs (Figure 4T and 4U).

### *In vivo* labeling of DCs and trafficking to peripheral lymph nodes

**Figure 5:**
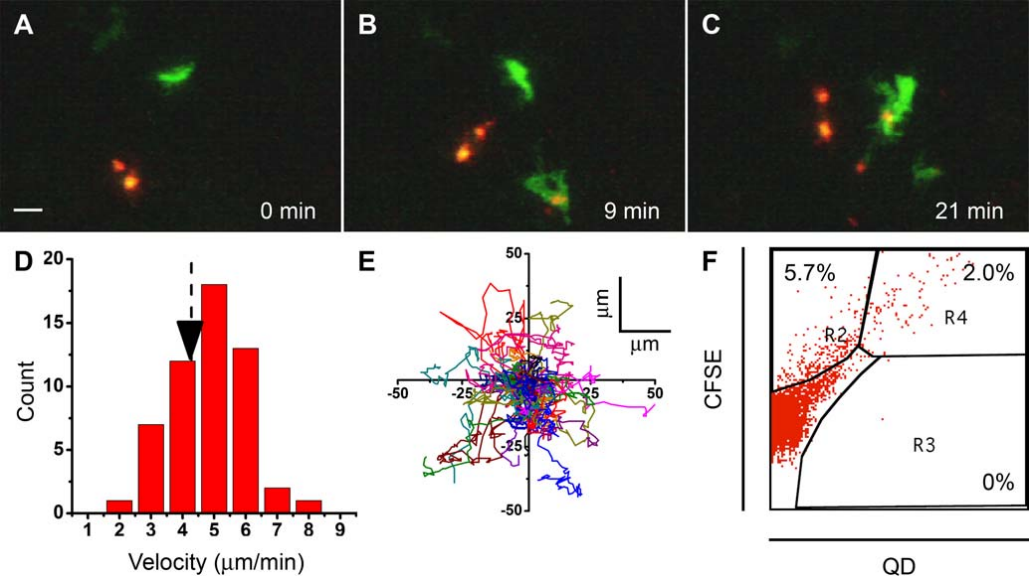
QD-labeled DCs visualized *in situ* in murine lymph nodes. (A–C) Sequential two-photon images of *in situ*-labeled DCs inside draining lymph nodes 24 hr after subcutaneous injection of CFSE (green) and QD (red) included in CFA. The time elapsed (min) after beginning imaging is indicated. The images were taken from Video S8. Scale bar = 20 µm. (D) Velocity distribution of DCs in lymph nodes. Arrow indicates average velocity (4.3±1.2 µm/min). (E) Trajectories of DCs in lymph nodes, normalized to their starting coordinates. (F) Profiles of CFSE^+^ and QD^+^ cells in draining lymph. Percentage of cells (except double negatives) have been indicated on the figure.

Based on our *in vitro* results and previous *in vivo* labeling of DCs with cell tracker dyes at an adjuvant depot [10], we anticipated that skin-resident DCs would take up subcutaneously injected QDs and subsequently traffic into draining lymph nodes. We injected mice subcutaneously with QD in complete Freund's adjuvant, either in eYFP-CD11c reporter mice or in BALB/c mice together with CFSE, and evaluated subsequent QD together with YFP or CFSE fluorescence in draining lymph nodes. At 4 hr, a time too early for skin-draining DCs to arrive in draining lymph nodes, QDs were observed in the lymph-node subcapsular sinus, but not inside the cortex (Figure S4). This is expected, as streptavidin-conjugated QDs have a size ∼2000 kD, larger than the approximate cutoff of ∼70 kD for free diffusion into the cortex from the subcapsular sinus [21]. QDs were also observed inside eYFP-expressing resident DCs lining the subcapsular sinus. One day after injection, DCs that had taken up either CFSE or QD, or both, were distributed within the T cell cortical region, and were excluded from the follicle (Figure 5A–5C). Flow cytometric analysis showed that QDs, as well as CFSE, preferentially labeled CD11c^+^ DCs (Figure S5A and S5B). Lymph node DCs labeled with QDs represent a mixture of migratory and resident DCs (Figure S5C), as indicated by the distribution of CD8α. Thus, consistent with our *in vitro* findings, skin-resident DCs near the site of injection take up QDs; and in addition, QDs that had migrated via lymphatics to the draining lymph node, are taken up by lymph node-resident DCs. The injected fluorophores also labeled a small percentage of GR-1^+^ granulocytes (Figure S5D and S5E). CFSE labeling of lymph node DCs was uniform through the cell body and processes, whereas QDs appeared compartmentalized in the cell body. DCs moved with an average velocity of ∼4 µm/min within the node, with individual DCs showing mean velocities distributed between 2–8 µm/min (Figure 5D). DCs frequently changed direction as they moved and did not exhibit any particular directional preference (Figure 5E). Movements were generally led by dynamic dendritic processes, followed by the cell body which often extended cytoplasmic “tails” (Video S8 and S9). Both CSFE and QD fluorescence could be resolved at depths down to ∼200 µm beneath the capsule, but only QD fluorescence remained visible below this, at depths up to 400 µm (data not shown). All QD^+^ cells were also CFSE^+^ (Figure 5F); thus QDs enabled deeper imaging by being brighter than CFSE under two-photon excitation.

### Antigen-conjugated QDs potently activate T cells *in vitro* and *in vivo*

**Figure 6:**
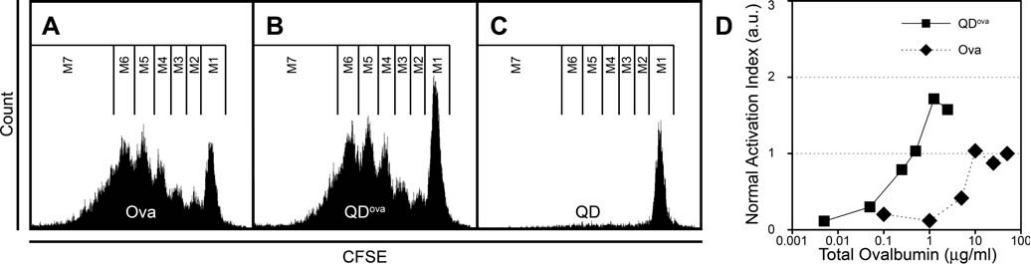
Activation of DO11.10 T cells *in vitro* by DCs pulsed with ovalbumin or QD^ova^. (A, B, and C) CFSE peaks representing consecutive cycles of division of CFSE-labeled T cells that were activated by DCs pulsed with 25 µg/ml ovalbumin (A), 5 nM QD^ova^ (2.5 µg/ml ovalbumin) (B), or QD alone (Streptavidin-conjugated QDs without ovalbumin) (C). Lines delineate sequential divisions M1–M7 used in Equation 1 to derive the activation index (see Methods). The amount of free ovalbumin present in the QD^ova^ sample was limited by serial dilution to <0.1 µg/ml during purification, and was insufficient to induce T cell activation and proliferation (not shown). (D) Normalized activation indices of T cell proliferation as functions of concentration of ovalbumin (diamonds) and QD^ova^ (squares). Data from at least 3 independent experiments for each of 6 different QD and QD^ova^ concentrations are presented.

To explore the potential use of QDs for particle-based antigen presentation, we measured the *in vitro* proliferation of TCR-transgenic ovalbumin-specific T cells in the presence of bone marrow-derived DCs pulsed with ovalbumin or ovalbumin-conjugated QDs (QD^ova^) (Figure 6A and 6B, respectively). Both treatments induced robust proliferation, whereas DCs pulsed with QDs alone failed to activate T cells (Figure 6C). Quantification of T cell proliferation using an activation index (*AI*; see Methods), revealed that QD^ova^ was ∼20 fold more effective in activating ova-specific T cells compared to equivalent amounts of free ovalbumin (Figure 6D). T cell activation plateaued at ovalbumin concentrations >50 µg/ml, and at QD^ova^ concentrations equivalent to >1.25 µg/ml ovalbumin. Moreover, the maximal proliferation was higher with QD^ova^ than with ovalbumin alone.

**Figure 7:**
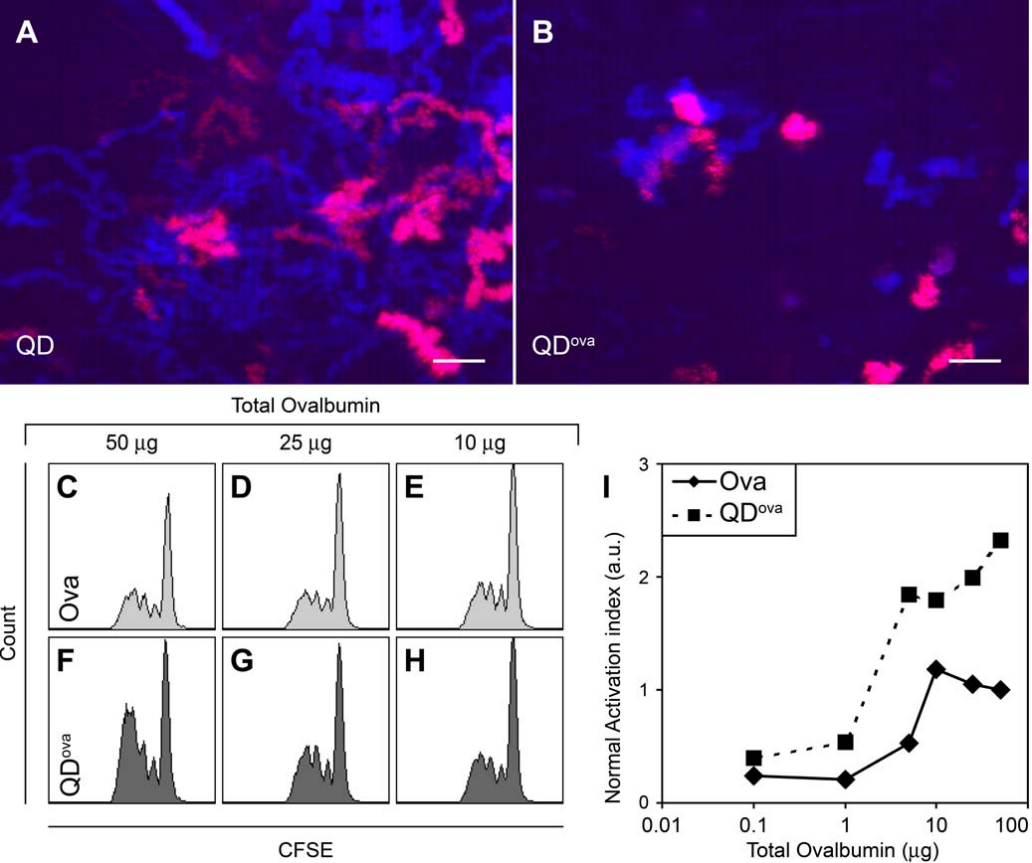
Activation of DO11.10 T cells *in vivo* by DCs pulsed with QD^ova^. Overlays of successive two-photon images of draining lymph nodes 8 hr after adoptive transfer of CMAC-labeled ova-specific T cells into mice immunized with QD (A) or QD^ova^ (B) included in 50 µl CFA. These images are single frames from Video S10 and S11, respectively. Scale bars = 20 µm. (C–H) Flow cytometry profiles showing activation of naïve T cells in draining lymph nodes of mice, into which ∼4×10^6^ CFSE-labeled ova-specific T cells were adoptively transferred, and which were subsequently s.c. injected in the lower flank with varying doses of free ovalbumin (C–E, light gray), or ovalbumin conjugated to QDs (dark gray) included in 50 µl CFA. (I) Normalized activation index as a function of total amount of ovalbumin delivered either unconjugated (diamonds) or conjugated with QDs (squares). Data are representative of at least 3 independent experiments.

We examined dynamic T cell / DC interactions in mice bearing adoptively transferred ova-specific T cells with and without immunization with QD^ova^. In mice injected with QDs without ova, T cells migrated freely, interacting transiently with DCs and covering a broad territory, as indicated by time overlays of superimposed frames (Figure 7A; Video S10). In contrast, clusters of T cells were observed surrounding QD^ova^-bearing DCs within draining lymph nodes from mice immunized with QD^ova^ (Figure 7B; Video S11). Time overlays showed that blue T cell tracks corresponded with red tracks of QD^ova^-bearing DCs. These T cell-DC clusters were observed at depths up to 150 µm. These results show that antigen-conjugated QD-labeled DCs can functionally engage in prolonged interactions with antigen-specific T cells to initiate an immune response.

To assess the ability of QD^ova^ to induce T cell proliferation *in vivo*, CFSE-labeled ova-specific T cells were adoptively into mice that were then immunized with either ovalbumin or QD^ova^. Upon immunization of these mice with ovalbumin and complete Freund's adjuvant (CFA), ova-specific T cells inside draining lymph nodes divided up to five times within 2 days (Figure 7C–7E; Figure S6A). Beginning ∼3 days after immunization, T cells that had divided were released into the circulation and were recovered from the spleen and non-draining lymph nodes (Figure S6). Ova-specific T cells also proliferated robustly upon immunization of mice with QD^ova^ (7F–7H). Assessment of relative *in vivo* T cell proliferation prior to T cell egress showed that QD^ova^ was >5 fold more effective than free ovalbumin in inducing ova-specific T cell proliferation (Figure 7I). T cells did not proliferate when injected with QDs (without antigen) or CFA alone (data not shown).

**Figure 8:**
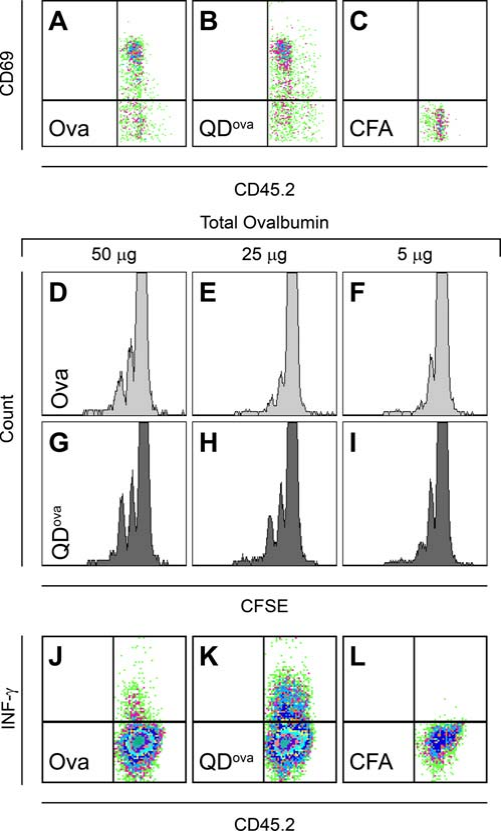
Activation of OT-II T cells *in vivo* by DCs pulsed with QD^ova^. (A, B, and C) CD69 upregulation in OT-II T cells following immunization with CFA containing either 50 µg free ovalbumin (A), QD^ova^ (B), or CFA alone (C). CD69 expression, evaluated 18 hr after immunization, was similar with free ova (∼60±7%, n = 3 experiments) and with QD^ova^ (∼65±6%, n = 3). (D–I) Proliferation of naïve OT-II T cells in draining lymph nodes of C57BL/6 mice s.c. injected with varying doses of either free ovalbumin (D–F, light gray), or ovalbumin conjugated to QDs (G–I, dark gray) included in 50 µl of CFA. Total amount of ovalbumin delivered in free form or conjugated to QDs is indicated. (J, K, and L) IFN-γ expression in mice adoptively transferred with OT-II T cells and immunized with CFA containing 50 µg free ovalbumin (J) or conjugated with QDs (K), or CFA alone (L). IFN-γ expression was evaluated 4 days after immunization. In three separate experiments, immunization with QD^ova^+CFA showed 2.4±0.6 greater upregulation of IFN-γ, compared to immunization with ovalbumin+CFA (p<0.05).

To evaluate the generality of T cell priming by QD^ova^, we examined a second transgenic TCR mouse also having sensitivity to ova but on an entirely different MHC background. To examine different stages of T cell activation, we evaluated CD69 expression and production of IFN-γ, in addition to cell proliferation (Figure 8). CD69 expression was up-regulated to a similar extent in mice immunized with either free ovalbumin+CFA or QD^ova^+CFA (Figure 8 A–C). QD^ova^ stimulated OT-II T cells to proliferate in C57BL/6 recipient mice (I-A^b^) more efficiently compared to free ovalbumin (Figure 8D–8I). Furthermore, production of IFN-γ was strongly enhanced in T cells inside draining lymph nodes of mice immunized with QD^ova^, compared to mice immunized with free ovalbumin (Figure 8K and 8J). Collectively, these results demonstrate that *in vivo* T cell priming is more effective using antigen-conjugated QDs compared to a standard immunization regimen with free antigen.

## Discussion

Our results demonstrate the utility of QDs for functional immuno-imaging of DCs and concomitant priming of T cell responses *in vitro* and *in vivo*. DCs were shown preferentially to acquire QDs by endocytosis; single-vesicle dynamics were tracked inside the cell as small endocytic vesicles jiggled and fused to form larger vesicles up to 1 µm in diameter. Single QDs were detected by electron microscopy inside endocytic vesicles that become increasingly electron dense during maturation. Rates of quantum dot uptake were reduced during toll-like receptor stimulation with LPS, consistent with a decrease in the rate of endocytosis during DC maturation. When included in a subcutaneous adjuvant depot, QDs were taken up by dendritic cells that migrated into the draining lymph node, where the bright and stable fluorescence of QDs facilitated two-photon imaging of DCs with detection limits at approximately twice the depth as other labels, although not revealing the dynamics of dendritic processes. Moreover, QDs bearing conjugated antigen were taken up by DCs and efficiently evoked T cell proliferative responses *in vitro* and *in vivo*.

DCs continually endocytose substances from the surrounding medium [2]. Previously, QDs have been used for real-time measurement of receptor-mediated endocytosis in transfected CHO cells [22]. Human DCs were recently reported to take up QDs conjugated to a ligand for the specific DC-receptor DC-SIGN by endocytosis of QDs [15]. In our study, we show that DCs avidly take up unconjugated or antigen-conjugated QDs, but without a requirement for conjugated endocytic DC cell-surface receptor-specific ligands. At a subcellular level, QD-uptake by DCs was not dependent on microfilaments, since disruption of F-actin by cytochalasin D did not inhibit uptake, consistent with previous reports showing that cytochalasin D does not inhibit endocytosis in mammalian macrophages and leukocytes [23], [24]. Endocytosis of small particles (<100 nm) by mammalian macrophages have been shown to be insensitive to cytochalasin D [25], [26], and the hydrodynamic diameter of QDs (<20 nm) falls well below this cutoff. Moreover, cytochalasin is known to inhibit phagocytosis but not pinocytosis [27]. QD uptake by DCs was not completely inhibited by nocodazole, indicating the existence of a microtubule-independent mechanism of QD uptake. Previous evidence shows that nocodazole does not inhibit endocytosis in bone marrow-derived phagocytes [28]. In contrast, polymerization of cortical actin by calyculin A completely inhibited QD uptake by DCs. Similar inhibition of endocytosis by calyculin A has been previously observed in T cells [23]. Together, these results indicate that QDs are endocytosed by DCs mainly via pinocytosis, and the presence of free actin is necessary for QD uptake. Endocytosed QDs are compartmentalized by DCs into vesicles. These QD-containing endosomes alternated between periods of high velocity and stationary ‘jiggling’. Endosomes are known to traffic along microtubules and can interact with actin via motor proteins [29], 30. Thus, as expected, vesicular motion was arrested by microtubule disruption using nocodazole. The periods of jiggling likely represent interactions of QD-containing endosomes with actin while trafficking along microtubules. Moreover, the absence of free actin in calyculin A-treated DCs probably caused vesicles to move freely along microtubles for long distances (Video S7). Eventually, endocytosed QDs are retained by DCs inside lysosomes for at least 48 hr.

Previous studies to visualize DCs *in vivo* have employed conventional fluorophores and fluorescent proteins, including *in vivo* labeling with cell tracker dyes included in alum adjuvant mixture [10], *in vitro* labeling with subsequent cell transfer [11], [31], [32], microinjection of fluorophores into lymph node [33], and expression of YFP driven by a CD11c promoter element in transgenic mice [34]. Here, we show that QDs are selectively taken up by dendritic cells *in vivo*, and can be used as a fluorescent marker for antigen-bearing DCs that traffic to draining lymph nodes from the site of immunization. Moreover, QD-bearing DCs can be imaged to greater depth inside draining lymph nodes without phototoxicity to the tissue. Thus, although not revealing the dynamics of DC dendrites, QDs are superior to commonly used fluorophores in terms of imaging depth in intact tissue.

Particle-based antigen delivery systems are being investigated for the development of efficient DC-based vaccines [35], [36]. In addition to using QDs for imaging of DCs, we further demonstrate that antigen-conjugated QDs can induce robust DC-mediated CD4^+^ T cell proliferation both *in vitro* and *in vivo* in two different strains of mice. QD delivery enhances T cell priming, leading to a 5–20 fold shift in the dose required to stimulate proliferation and a strong enhancement of IFN-γ production, compared to a standard immunization using equivalent amounts of free antigen (ovalbumin). Since QDs are avidly endocytosed by DCs, conjugation to QDs likely increases the rate of antigen uptake by DCs as compared to free antigen. The ovalbumin bound to QD is subsequently processed by the DCs and presented to T cells, whereas the QDs themselves are not processed by lysosomal enzymes or trafficked further into the MHC class-II pathway, but are instead retained inside DCs. Concentrations (5–10 nM) of QDs sufficient to induce robust T cell proliferation did not produce any evident toxic effects in DCs observed over a period of 48 hr, and QD^+^ cells migrated to the lymph node 12–16 hr after subcutaneous injection, consistent with previous studies showing that QDs can be used for several days *in vivo* without deleterious effects [37], [38].

In conclusion, QDs can serve as multi-functional probes with unique advantages for both imaging studies employing correlated fluorescence and electron microscopy, and as a potent particle-based system for antigen delivery. QDs are versatile fluorophores that can be used to fine-tune important functional aspects of the immune system, in conjunction with correlated fluorescence and electron microscopy, and may have potential for use in immunotherapy and vaccine development.

## Methods

### Mice

BALB/c (I-A^d^), C57BL/6J (I-A^b^), and congenic CD45.1-expressing BL6/SJL (C57BL/6) mice were purchased from Jackson Laboratory. Ova peptide^323–339^/I-A^d^-specific TCR-transgenic DO11.10 mice (KJ1.26^+^) were progeny of homozygotic DO11.10 parents purchased from Jackson Laboratory. Ova peptide^323–339^/I-A^b^-specific TCR-transgenic OT-II mice were progeny of homozygotic OT-II parents purchased from Jackson Laboratory. Mice expressing enhanced yellow fluorescent protein (EYFP) on CD11c promoter were a kind gift from M. Nussensweig [34]. These mice were back-crossed for 10 generations to a C57BL/6 background. Mice were housed in a pathogen-free animal facility and all procedures were performed in accordance with protocols approved by the institutional animal care and use committee of UCI.

### Conjugation of ovalbumin to quantum dots

Ovalbumin was biotinylated using EZ-Link™ Sulfo-NHS-Biotin (Pierce™). Excess biotin and salts were separated by gel filtration using D-Salt™ dextran desalting columns (Pierce™). Biotinylation of ovalbumin was verified by Western blotting. Quantum dot (QD) 655-streptavidin conjugates (100 nmol; Quantum Dot Corp./Invitrogen™) were mixed with biotinylated ovalbumin at a molar ratio of 1∶10 in 2 ml PBS at 6°C for 2 hr. Excess ovalbumin was removed by at least 4 rounds of ultrafiltration using Amicon Ultra™ 4 100,000 MWCO ultrafiltration units (Millipore Corp.). Each QD-streptavidin conjugate contains an average of 10 molecules of surface-bound streptavidin (Quantum Dot Corp.). Thus, a 1 nM solution of QD-streptavidin conjugates bound to biotinylated ovalbumin, as used for priming DCs *in vitro*, contains 10 nM ovalbumin, which is equivalent to 0.5 µg/ml of ovalbumin.

### *In vitro* dendritic cell culture

Dendritic cells were cultured from tibial and femoral bone marrow extracts of 8–12 week old mice as described [39]. Briefly, the bone marrow extracts were cultured in non TC-treated polystyrene culture dishes (Corning™) using IMDM (Lonza™) substituted with 10% fetal calf serum (FCS; Hyclone Inc.), ∼1,000 units/ml (20 ng/ml) recombinant mouse granulocyte/macrophage colony stimulating factor (GM-CSF; Pharmingen™), 100 units/ml penicillin, and 100 µg/ml streptomycin. DCs were harvested between six and ten days of culture and used in experiments. For flow cytometry of QD^+^ cells, harvested DCs were incubated in QD-containing medium (2 nM QD in RPMI substituted with 10% FCS), for 30 min at 37°C, and subsequently labeled with FITC-conjugated anti-mouse CD11c (Pharmingen™), PE-conjugated anti-mouse CD11b (Ebioscience™)To induce maturation, DCs were incubated with 1 µg/ml lipopolysachharide (LPS) (Sigma-Aldrich Inc.) for 12–16 hr, and maturation was verified by upregulation of MHC class-II (PE-conjugated anti-mouse MHC-II; Pharmingen™) and CD86 (PE-conjugated anti-mouse CD86; Pharmingen™).

### *In vitro* imaging of DCs, quantification of QD uptake, and vesicular dynamics

For *in vitro* imaging, bone marrow-derived DCs were plated on cover glass chambers, and incubated with 2 nM QD-containing medium. Unless otherwise mentioned, temperature was maintained at 37°C by perfusing temperature-controlled PBS below the cover slip. For assessment of QD-toxicity in DCs propidium iodide (Calbiochem™) staining was used. Fluorescence and DIC images were acquired with a Zeiss Axiovert 35 microscope using a 40× 1.30 n.a. oil-immersion objective, equipped with a temperature controller. Higher resolution images were acquired with a custom-built video-rate two-photon microscope based on a Olympus BX50 confocal laser-scanning system, equipped with a titanium-sapphire femtosecond laser (Tsunami, Spectra-Physics) tuned to 780 nm, photomultiplier tubes for detection, and a 60× 1.10 NA water-immersion objective as previously described [40]. Every image acquired was an average of 15 video-rate frames (acquired at a rate of 30 frames/s using Metamorph™ software (Universal Imaging / Molecular Devices) and Video Savant (IO Industries™). Metamorph™ was used to create time-lapse videos from sequential two-photon images, and to observe and analyze QD uptake and the trafficking of QD-containing endosomes inside DCs. Images were acquired at a spatial resolution of 4.5 pixels/µm using 110 mW mean laser power for quantification of QD uptake, and at a spatial resolution of 12 pixels/µm for measurement of kinetics of QD-containing vesicles. For evaluation of antigen uptake in immature and mature DCs, QD-fluorescence intensity was measured over time. After background subtraction, intensities were normalized to the intensity measured 400 s after incubation of DCs in QD-containing medium. The linear slopes of intensities were measured to quantify rate of uptake of QDs by DCs. Velocities and displacements of individual vesicles were measured from unprocessed sequential two-photon images taken at intervals of 700 ms. Normalized displacements of individual vesicles were plotted to depict trajectories. For experiments involving temperature-variation of vesicular dynamics, temperatures were varied between 23°C and 37°C. Q_10_ was calculated as Q_10_ = 10^ΔlogV^, where ΔlogV is the change in the value of log_10_velocity for a 10°C rise in temperature. To observe the effects of cytoskeletal inhibitors on uptake of QDs, bone marrow-derived DCs were labeled with 10 µM CFSE (Invitrogen™) for 15 min at 37°C, and subsequently treated with calyculin A (Sigma-Aldrich™; 200 nM for 20 min), cytochalasin D (Sigma-Aldrich™; 1 µM for 1 hr), or nocodazole (Sigma-Aldrich™; 1 µM for 1 hr). These DCs were then incubated with 2 nM QD-containing medium at 37°C in the maintained presence of each of the reagents at the same concentration as used for pre-treatment. Using Metamorph™ QD-fluorescence and CFSE-fluorescence were measured within regions containing >5 cells after 10 min of incubation. Fluorescence intensity was normalized to intensity of CFSE and has been presented as a ratio of QD-fluorescence intensity of untreated control DCs. To observe the effects of cytoskeletal inhibitors on vesicular motion, bone marrow-derived DCs were first allowed to take up QD for 30 min at 37°C, and subsequently treated with calyculin A (200 nM), cytochalasin D (1 µM), or nocodazole (1 µM) during imaging. For lysosomal co-localization, harvested DCs were cultured overnight in glass-bottom culture dishes (Mattek™), incubated with 2 nM QD-containing medium for varying times at 37°C. Subsequently, DCs were fixed with 2% paraformaldehyde in PBS for 30 min at 4°C, and then stained with 20 µg/ml FITC-conjugated anti-mouse LAMP-2 (Pharmingen) for 2 hr at 4°C to stain lysosomes. DCs were washed with PBS, dehydrated in 100% ethanol, mounted using Vectashield™ hardest mounting medium (Vector Labs), and left overnight at 4°C before imaging. To observe the effects of cytoskeletal inhibitors on F-actin organization and QD-uptake, bone marrow-derived DCs were treated with cytochalasin D, nocodazole, or calyculin A, and then incubated with 2 nM QD-containing medium for 15 min at 37°C in the maintained presence of these reagents, as described earlier. Subsequently these DCs were plated on glass-bottom culture dishes, fixed with 4% paraformaldehyde and stained with 50 µg/ml FITC-phalloidin for 1 hr at room temperature. These samples were washed with PBS, dehydrated in 100% ethanol, mounted using Vectashield™ hardest mounting medium (Vector Labs), and left overnight at 4°C before imaging. Fixed DCs were imaged using a Zeiss LSM-510 confocal microscope equipped with a Zeiss™ PlanApochromat™ 63× 1.40 N.A. oil-immersion objective.

### *In vivo* imaging of DC and determination of DC phenotype

BALB/c mice were s.c. injected in lower flank with 100 nmol CFSE (green) and 20 pmol QD (red) included in 50 µl of complete Freund's adjuvant (CFA, Sigma™). Draining inguinal and brachial nodes from injected mice were harvested 24 hr later and observed using two-photon microscopy as described [10]. Briefly, the nodes were placed on plastic cover slips (Fisher™) using Vetbond™ (3M™), perfused with oxygenated RPMI (Hyclone Inc.) maintained at 37°C, and observed using two-photon microscopy. Images of several z sections (each an average of 15 frames acquired at 30 frames/s) at varying distances of separation were combined to create individual 3D time-points using Metamorph™. For locating DCs with respect to B cell follicles, mice were adoptively transferred with MACS™ magnetic separation chromatography-enriched (Miltenyi Biotec.) B cells from BALB/c mice labeled with 25 µM 7-amino-4-chloromethylcoumarin (CMAC, blue; Molecular Probes™ / Invitrogen™) for 45 min at 37°C; 24 hr later these mice were s.c. injected with 100 nmol CFSE (green) and 20 pmol QD (red) included in 50 µl CFA. 24 hr after injection, draining lymph nodes were harvested for two-photon imaging. DCs were recovered from lymph nodes for flow cytometry, as described [41]. Briefly, lymph nodes from injected mice were harvested 24 hr after s.c. injection of dye-CFA mixture, digested with 1 mg/ml Collagenase, Type IV (Worthington Biochemical) for 30 min at 37°C in DMEM substituted with 2% FBS. Cells were subsequently labeled with PE-conjugated anti-mouse CD11c (Ebioscience™), PE-conjugated anti-mouse GR-1 (Ebioscience™), or PE-conjugated anti-mouse CD8α (Ebioscience™), fixed with 2% paraformaldehyde in PBS, and analyzed by flow cytometry using Facscalibur™ flow cytometer (BD Biosciences).

### Electron microscopy

Harvested DCs were cultured overnight in glass-bottom culture dishes (Mattek™), incubated with 2 nM QD-containing medium for varying times at 37°C, and processed as described [23]. Briefly, DCs were fixed with 2% glutaraldehyde (Electron Microscopy Sciences) in 0.1 M sodium cacodylate (Ted Pella™) buffer (pH 7.3) for 20 min, washed with 0.1% cacodylate buffer, and postfixed with 1% osmium tetroxide (Electron Microscopy Sciences) solution for 30 min. Subsequently, the DCs were counterstained with 4% uranyl acetate (Electron Microscopy Sciences) for 30 min, washed with distilled water, dehydrated in 100% ethanol, and embedded in Durcupan™ ACM resin (Fluka™).

### *In vitro* T cell proliferation assay

DCs (∼4×10^5^ cells) were incubated with varying concentrations of ovalbumin or QD^ova^ in U-bottom polystyrene tubes (Fisher Scientific™). Antigen-pulsed DCs were incubated 6 hr later with 1 µg/ml LPS for 12–16 hr. CD4^+^ T cells were purified from spleen and lymph nodes of DO11.10 mice by depleting CD8^+^ T cells, B cells, NK cells, DCs, macrophages, granulocytes, and RBCs using MACS™ magnetic cell separation kit (Miltenyi Biotec.). Subsequently, the T cells were labeled with 4 µM carboxyfluorescein diacetate succinimidyl ester (CFSE) (Molecular Probes™/Invitrogen™) at 37°C for 10 min and ∼2×10^6^ cells (5 T cells per DC) were put into each U-bottom tube containing antigen-pulsed or control DCs in T cell medium (RPMI, 10% FCS, 1 mM sodium pyruvate, 1% non-essential amino acids, 1% l-glutamine, 50 µM β-mercaptoethanol, 100 units/ml penicillin, and 100 µg/ml streptomycin). After 72–96 hr at 37°C T cells were harvested and stained using PE-conjugated anti-mouse DO11.10 clonotypic T cell receptor (KJ1-26) antibody (Pharmingen™). CFSE counts were gated on KJ1-26^+^ cells and CFSE dilution was analyzed using flow cytometry to measure proliferation due to T cell activation. T cell activation was measured in terms of an activation index (*AI*) using Equation 1, derived as described in Methods S1. *M_1_* through *M_7_* are the cumulative counts of T cells within the gates M_1_ through M_7_, shown in Figure 6A–6C. For comparison and evaluation of varying antigen doses, the activation indices were normalized to the activation index of 50 µg/ml ovalbumin.
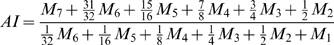
(1)


### *In vivo* T cell proliferation assay

T cells from DO11.10 (ova peptide^323–339^/I-A^d^) or OT-II mice (ova peptide^323–339^/I-A^b^) were enriched using MACS™ as described above and labeled with 4 µM CFSE at 37°C for 15 min. ∼4×10^6^ CFSE-labeled cells were adoptively transferred into BALB/c (for DO11.10 T cells) or C57BL/6 (for OT-II T cells) recipients, and allowed to equilibrate for 24 hr. Subsequently the recipient mice were s.c. injected with varying dose of ovalbumin or QD^ova^, or QDs included in 50 µl CFA, or CFA alone in the lower flank. Lymphocytes were recovered from lymph nodes and spleens harvested 2–4 days later. Harvested lymphocytes from BALB/c recipients were stained with PE-conjugated anti-mouse KJ1-26 antibody (Pharmingen) and analyzed for proliferation using flow cytometry by gating on KJ1-26. Harvested lymphocytes from C57BL/6 recipients were stained with PE-conjugated Vβ5.1/5.2 and APC-conjugated Vα2, and analyzed for proliferation using flow cytometry by gating on Vβ5.1/5.2 and Vα2. T cell activation was measured in terms of activation index (*AI*) using Equation 1 with five terms.

### Imaging ova-specific T cell-DC interactions

T cells from DO11.10 mice were labeled with blue CMAC (20 µM at 37°C for 45 min) or green CFSE (10 µM at 37°C for 15 min) and ∼4×10^6^ cells were adoptively transferred into BALB/c recipients. 24 hr later mice were s.c. injected on one flank with QD^ova^ (containing 50 µg ovalbumin), and on the opposite flank with QD included in 50 µl complete Freund's adjuvant. Lymph nodes were harvested 18 hr later and imaged using two-photon microscopy, as described earlier. In separate experiments, mice were immunized with QD^ova^ or QD, and 12 hr later adoptive transferred with blue CMAC-labeled T cells. Lymph nodes were harvested 8 hr after adoptive transfer of T cells and imaged using two-photon microscopy.

### Assessment of IFN-γ production in T cells in immunized mice

T cells from OT-II mice (expressing CD45.2) were enriched as described above, and labeled with 4 µM CFSE at 37°C for 15 min. ∼2×10^6^ CFSE-labeled cells were adoptively transferred into congenic CD45.1-expressing BL6 recipients, and allowed to equilibrate for 24 hr. Subsequently, the recipient mice were s.c. injected with 50 µg ovalbumin or QD^ova^, in both cases in 50 µl CFA. Lymphocytes were harvested from draining inguinal lymph nodes or control mesenteric lymph nodes 4 days after immunization. Harvested lymphocytes were re-stimulated with PMA and ionomycin *in vitro* for 6 hr. For the last 2 hr BD™ Golgistop™ was added to the medium. Subsequently, lymphocytes were stained with PE-conjugated anti-mouse CD45.2 (Ebioscience), fixed using BD™ cytofix-cytoperm™ cytokine staining kit, and labeled with APC-conjugated anti-mouse IFN-γ (Ebioscience) or isotype control. Labeled cells were assessed for IFN-γ production by gating on CD45.2^+^ cells.

## Supporting Information

Methods S1 Equipment and Settings, Calculation of T cell activation index (AI)
(0.07 MB DOC)

Figure S1 Quantum dots are not toxic to DCs. DCs were incubated with 10 nM QD 525-Streptavidin conjugate for 48 hours, stained with 20 µg/ml of propidium iodide, and assessed for toxicity using flow cytometry. Histograms showing propidium iodide staining in untreated cells (green), QD-treated cells (red), and cells fixed with 2% glutaraldehyde (black). Isotype control has been shown in gray. While fixed DCs were ∼100% PI+, ∼15–20% of QD-treated DCs were PI+, comparable to untreated DCs. Thus QDs did not show toxicity at a concentration of 10 nM, higher than any of the concentrations used in our experiments
(0.37 MB TIF)

Figure S2 Flow cytometry profile of cells that endocytosed QDs. Bone marrow-derived cells were incubated for 30 min with 2 nM QD at 37 µC, then washed and stained with (A) FITC-conjugated anti-CD11c or (B) FITC-conjugated anti-CD11b, and analyzed by flow cytometry by gating on the corresponding markers as shown in figure. ∼70% QD+ cells were CD11c+, and ∼75% QD+ cells were CD11b+.
(0.39 MB TIF)

Figure S3 Confocal images showing effects of cytoskeletal inhibitors on F-actin organization and QD-uptake by DCs. DCs were either (A–C) untreated, or treated with (D–F) cytochalasin D (1 µM for 1 hr at 37°C), (G–I) nocodazole (1 µM for 1 hr at 37°C), or (J–L) calyculin A (200 nM for 20 min at 37°C), and incubated with media containing 2 nM QD (red) for 20 min in the maintained presence of these reagents. Subsequently, these DCs were fixed and stained with FITC-phalloidin to label F-actin (green). Confocal images are consistent with results obtained using real-time two-photon imaging. Scale bar = 10 µm.
(3.51 MB TIF)

Figure S4 QDs in subcapsular sinus of draining lymph nodes. DCs were labeled in situ by subcutaneously injecting EYFP-CD11c mice with QDs included in 50 µl CFA. Draining lymph nodes were harvested 4 hrs later for imaging. Subcapsular fibers appear blue due to second harmonics. (A) QDs (red) are trapped inside vessels in the capsule and are presumably taken up by subcapsular DCs (green) and macrophages. (B, C) Z sectional views of the capsule show that QDs are not yet present inside the node below the capsule. Scale bar = 20 µm.
(5.15 MB TIF)

Figure S5 Phenotypic profile of DCs observed by flow cytometry. Phenotypic markers are indicated on the Y-axis of each plot. Distribution of QD+ and CFSE+ cells (percent) for each phenotypic marker is shown on the graph.Note the similarity in distribution of QD+ and CFSE+ cells for each phenotypic marker.
(0.86 MB TIF)

Figure S6 In vivo T cell response at different times following immunization. BALB/c mice were adoptively transferred with ∼4×10^6^ CFSE-labeled DO11.10 T cells, and subsequently immunized with 50 µg of ovalbumin included in 50 µl CFA. Control and draining lymph nodes, and spleens were harvested (A–C) 2 days, (D–F) 3 days, or (G–I) 4 days after injection, and analyzed for T cell activation using flow cytometry. Day 2 (just prior to egress of activated T cells) was chosen as the time point for analysis of T cell activation.
(0.32 MB TIF)

Video S1 QD-labeled DCs *in vitro*
(5.34 MB AVI)

Video S2 QD-uptake by DCs *in vitro*
(7.53 MB AVI)

Video S3 Fusion of QD-containing vesicles inside DCs
(2.56 MB AVI)

Video S4 Vesicles jiggling inside a single DC
(8.19 MB AVI)

Video S5 Effect of cytochalasin D on vesicular motion
(7.58 MB AVI)

Video S6 Effect of nocodazole on vesicular motion
(6.22 MB AVI)

Video S7 Effect of calyculin A on vesicular motion
(10.30 MB AVI)

Video S8 In situ QD and CFSE-labeled DCs inside draining lymph nodes
(4.61 MB AVI)

Video S9 Localization of in situ-labeled DCs inside draining lymph nodes relative to B cell follicles
(4.15 MB AVI)

Video S10 Ova-specific T cells alongside QD-labeled DCs inside lymph nodes
(7.69 MB AVI)

Video S11 Ova-specific T cells cluster around QD^ova^-labeled DCs inside lymph nodes
(4.94 MB AVI)
