# Supplementary material for: Quantum Dots for Tracking Dendritic Cells and Priming an Immune Response In Vitro and In Vivo
Source: PLoS One. 2008 Sep 29;3(9):e3290. doi: 10.1371/journal.pone.0003290 (PMC2538605; doi:10.1371/journal.pone.0003290)
Supplement: Methods S1 — Equipment and Settings, Calculation of T cell activation index (AI) (0.07 MB DOC) [file pone.0003290.s001.doc]

**Online Supporting Information**

**Equipment and Settings**

Brightfield and two-photon images were acquired as summarized in Methods. For brightfield imaging, a Zeiss Axiovert™ 35 microscope equipped with a 40× 1.30 n.a. oil-immersion objective, and a temperature controller was used. Digital images were acquired using a Photometrics™ CoolSnapHQ™ CCD camera at an exposure time of 500 ms per frame. Two-photon images were acquired using an Olympus BX50™ microscope equipped with a custom video-rate laser-scanning system, a titanium-sapphire femtosecond laser, and photomultiplier detectors. A 60× 1.10 N.A.. objective was used for imaging single-vesicle dynamics, and a 20× 0.95 N.A.. water-immersion objective was used for *in vivo* imaging inside murine lymph nodes.

Figure 1A is a single frame from Video S1 acquired using Brightfield and epifluorescence imaging, at the rate of 6 frames/min. Figure 1B was acquired using two-photon imaging, as an average of 15 frames acquired at a rate of 30 frames/s.

Figure 2A-2C and 2G-2I are frames from Videos S2 and S3, respectively, acquired using two-photon imaging at the rate of 30 mainframes/s, with each panel showing an average of 15 frames. . Raw image stacks were analyzed using Metamorph™ software to obtain mean fluorescence intensity at each time point, and the analyzed data are presented using Origin™ software in Figure 2N. Figure 2J is a single frame from Video S4, acquired as in Figure 1B. Gamma correction and contrast enhancement were applied for clarity. Raw image stacks were analyzed using Metamorph software to track individual vesicles, and the analyzed data (2K-2M) are presented using Origin™ software.

Figure 3A-3O were acquired using a Zeiss™ LSM 510 meta confocal microscope. Figure 3P-3U were acquired at the National Center for Microscopy and Imaging Research, University of California, San Diego, Contrast was enhanced for clarity.

Figure 4A-4C are frames from Video S8, acquired using two-photon microscopy. Frames are 200 μm × 150 μm. Depths (Z-axis) of 50 μm at 2.5 μm steps were imaged. For each time point Z axis frames have been superposed to convert a 3D image to a 2D representation using Metamorph™ software. Velocities and displacements were analyzed using Metamorph and are plotted by Origin™ software in Figure 4D and 4E, respectively. Figure 4F-4L were acquired using a BD FacSCAN™ flow cytometer, and analyzed using BD Cellquest™ software.

Figure 5A-5C were acquired using a BD FacSCAN™ flow cytometer, and using BD Cellquest™ software. Figure 5D was plotted using Microsoft Excel™ software.

Figure 6A-6B are frames from Video S10 and S11 respectively, acquired as described in Figure 4A-4D. Gamma correction has been performed for clarity. 6C-6H were acquired using a BD FacSCAN™ flow cytometer, and analyzed and presented using BD Cellquest™ software. Figure 6I was plotted using Microsoft Excel™ software.

**Calculation of T cell activation index (*AI*)**

Firstly, we consider each CFSE peak as a stage, and number the first stage as Stage 1, and subsequent stages as stage 2, 3, etc. Activation index (*AI*) is calculated by dividing the total number of observed divisions by the total number of cells input (Equation I). It is assumed that due to adhesiveness, loss, apoptosis, etc., a certain fraction of cells lost is reflected in the analysis. Thus Equation II gives the number of cells measured at each Stage N. Now we can assume that the number of cells at every Stage N, were derived from $C_{N}/2$cells from Stage N-1, which in turn were derived from $C_{N}/4$ cells from Stage N-2, and so on and so forth. Thus Equation III gives the total number of input cells corresponding to the total number of cells at any Stage N. Therefore counting for every stage, the total number of input cells is given by Equation IV. Now the number of cells at every Stage N, were derived from $C_{N}/2$divisions from Stage N-1, which in turn were derived from $C_{N}/4$ divisions from Stage N-2, and so on and so forth. Thus Equation V gives the total number of divisions $D_{N}$ leading to production of cells at any Stage N, which can be reduced to Equation VI. Therefore counting for every stage, the total number of divisions is given by Equation VII. Thus substituting for total number of divisions (Equation VII) and total number of cells (Equation IV) in Equation I, we get *AI* as in Equation VIII. For simplicity we assume that the loss is identical at every stage in a given assay, i.e. $p_{1}=p_{2}=p_{3}=\cdots=p_{N}$. Thus Equation VIII reduces to Equation IX. Within limits of experimental error we can assume that $\left(1/2\right)^{N}=0forN\geq6$. Thus Equation IX further reduces to Equation X. Now, each of the terms in Equation X can be attributed to the actual gate counts, obtained using histogram analysis using Cellquest™. The gates have been shown in Figure 6. Thus *AI* can be calculated using Equation XI, which is derived by substituting each term of Equation X by the corresponding gate count.

Equation I

$$AI=\frac{\#Divisions}{\#Input\_Cells}$$

Equation II

$\#Cells\_at\_Stage\_N=C_{N}(1+p_{N})$ [$C_{N}$ is the observed count, and $p_{N}$ is the assumed loss]

Equation III

$$\#Input\_corresponding\_to\_Stage\_N=\frac{C_{N}(1+p_{N})}{2^{N-1}}$$

Equation IV

$$\#Input\_Cells=C_{N}(1+p_{N})\frac{1}{2^{N-1}}+\cdots+C_{3}(1+p_{3})\frac{1}{2^{2}}+C_{2}(1+p_{2})\frac{1}{2}+C_{1}(1+p_{1})$$

Equation V

$$D_{N}=C_{N}(1+p_{N})\frac{1}{2^{N-1}}+\frac{1}{2^{N-2}}+\cdots+\frac{1}{2}$$

Equation VI

$$\therefore D_{N}=C_{N}(1+p_{N})\left(1-\frac{1}{2^{N-1}}\right)$$

Equation VII

$$\#Divisions=C_{N}(1+p_{N})\left(1-\frac{1}{2^{N-1}}\right)+\cdots+C_{3}(1+p_{3})\left(1-\frac{1}{2^{2}}\right)+C_{2}(1+p_{2})\left(1-\frac{1}{2}\right)$$

Equation VIII

$$AI=\frac{C_{N}(1+p_{N})\left(1-\frac{1}{2^{N-1}}\right)+\cdots+C_{3}(1+p_{3})\left(1-\frac{1}{2^{2}}\right)+C_{2}(1+p_{2})\left(1-\frac{1}{2}\right)}{C_{N}(1+p_{N})\frac{1}{2^{N-1}}+\cdots+C_{3}(1+p_{3})\frac{1}{2^{2}}+C_{2}(1+p_{2})\frac{1}{2}+C_{1}(1+p_{1})}$$

Equation IX

$$AI=\frac{C_{N}\left(1-\frac{1}{2^{N-1}}\right)+\cdots+C_{3}\left(1-\frac{1}{2^{2}}\right)+C_{2}\left(1-\frac{1}{2}\right)}{C_{N}\frac{1}{2^{N-1}}+\cdots+C_{3}\frac{1}{2^{2}}+C_{2}\frac{1}{2}+C_{1}}$$

Equation X

$$AI=\frac{\sum_{7}^{\infty} C_{N}+\frac{31}{32}C_{6}+\frac{15}{16}C_{5}+\frac{7}{8}C_{4}+\frac{3}{4}C_{3}+\frac{1}{2}C_{2}}{\frac{1}{32}C_{6}+\frac{1}{16}C_{5}+\frac{1}{8}C_{4}+\frac{1}{4}C_{3}+\frac{1}{2}C_{2}+C_{1}}$$

Equation XI

$$AI=\frac{M_{7}+\frac{31}{32}M_{6}+\frac{15}{16}M_{5}+\frac{7}{8}M_{4}+\frac{3}{4}M_{3}+\frac{1}{2}M_{2}}{\frac{1}{32}M_{6}+\frac{1}{16}M_{5}+\frac{1}{8}M_{4}+\frac{1}{4}M_{3}+\frac{1}{2}M_{2}+M_{1}}$$
