# Supplementary figures and images for: Quantum Dots for Tracking Dendritic Cells and Priming an Immune Response In Vitro and In Vivo
Source: PLoS One. 2008 Sep 29;3(9):e3290. doi: 10.1371/journal.pone.0003290 (PMC2538605; doi:10.1371/journal.pone.0003290)

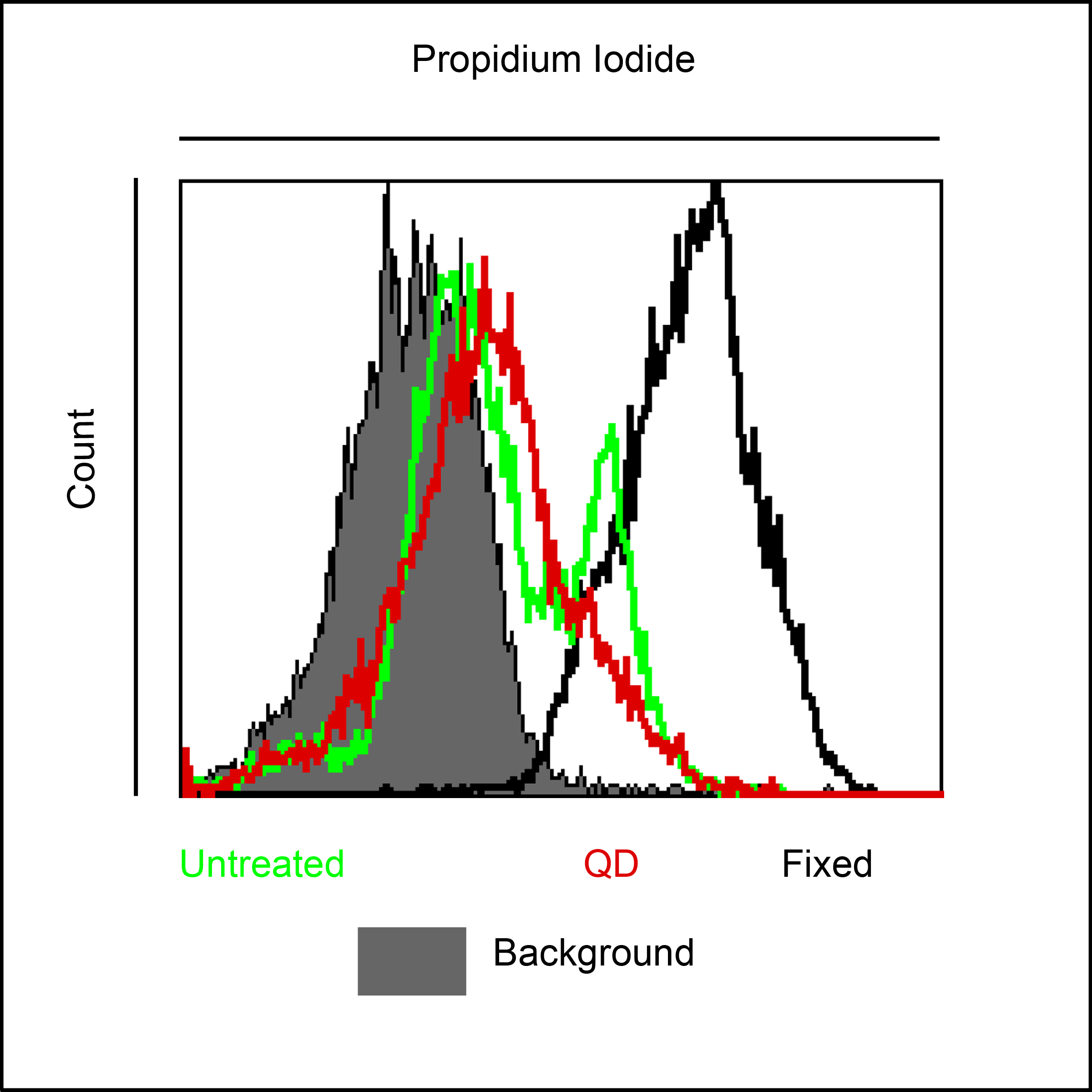

Supplement: Figure S1 — Quantum dots are not toxic to DCs. DCs were incubated with 10 nM QD 525-Streptavidin conjugate for 48 hours, stained with 20 µg/ml of propidium iodide, and assessed for toxicity using flow cytometry. Histograms showing propidium iodide staining in untreated cells (green), QD-treated cells (red), and cells fixed with 2% glutaraldehyde (black). Isotype control has been shown in gray. While fixed DCs were ∼100% PI+, ∼15–20% of QD-treated DCs were PI+, comparable to untreated DCs. Thus QDs did not show toxicity at a concentration of 10 nM, higher than any of the concentrations used in our experiments (0.37 MB TIF) [file pone.0003290.s002.tif]

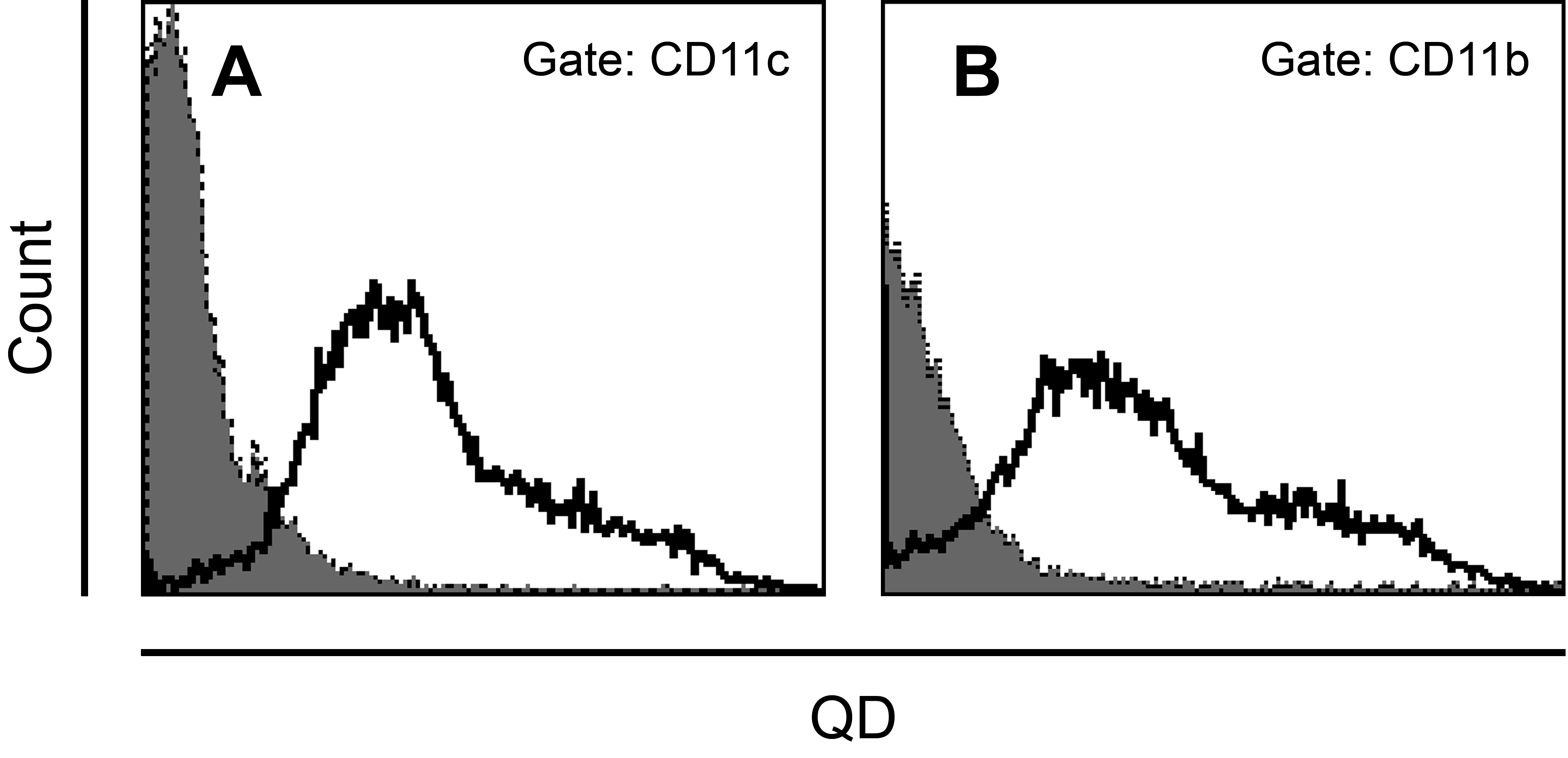

Supplement: Figure S2 — Flow cytometry profile of cells that endocytosed QDs. Bone marrow-derived cells were incubated for 30 min with 2 nM QD at 37 µC, then washed and stained with (A) FITC-conjugated anti-CD11c or (B) FITC-conjugated anti-CD11b, and analyzed by flow cytometry by gating on the corresponding markers as shown in figure. ∼70% QD+ cells were CD11c+, and ∼75% QD+ cells were CD11b+. (0.39 MB TIF) [file pone.0003290.s003.tif]

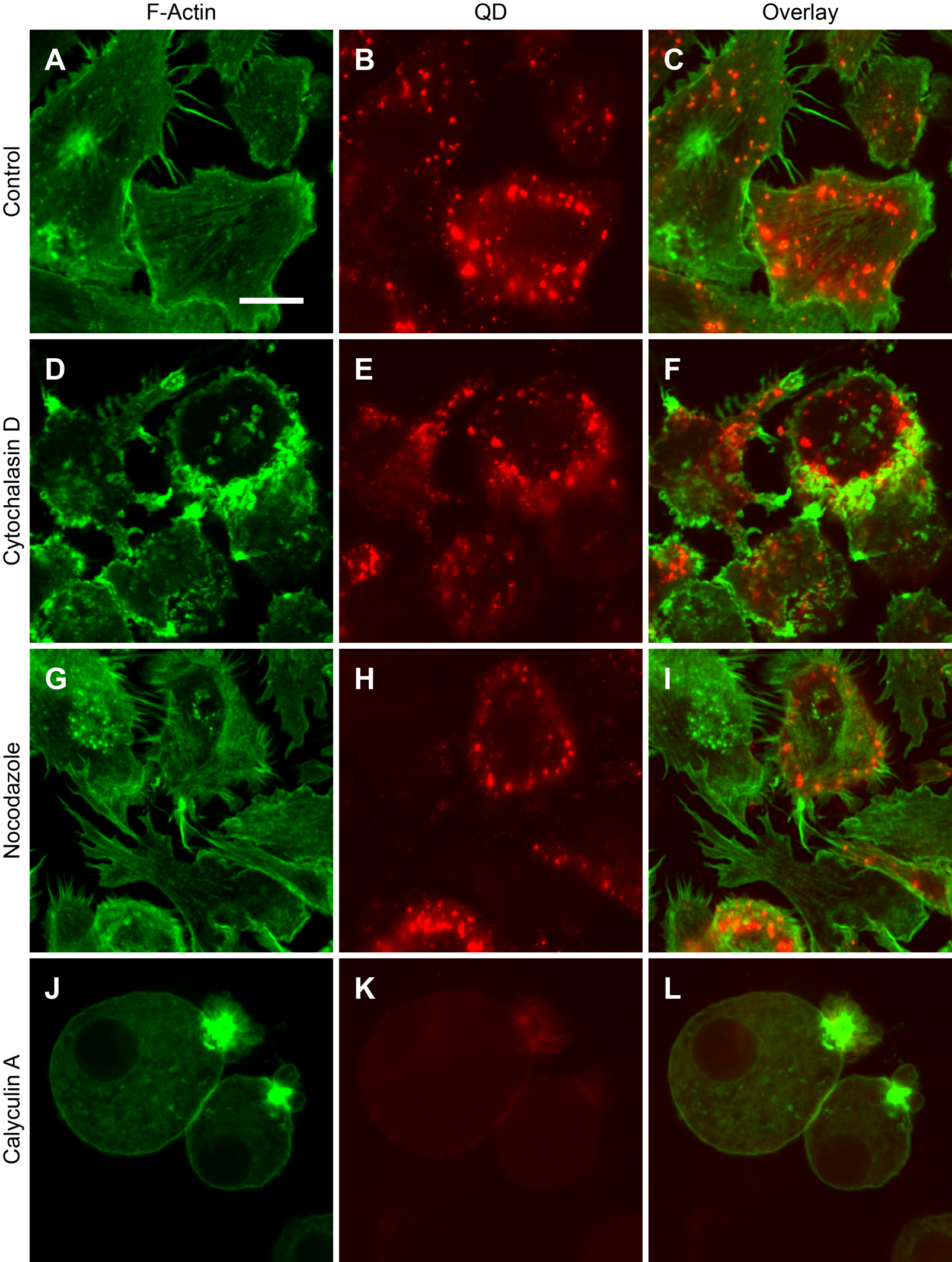

Supplement: Figure S3 — Confocal images showing effects of cytoskeletal inhibitors on F-actin organization and QD-uptake by DCs. DCs were either (A–C) untreated, or treated with (D–F) cytochalasin D (1 µM for 1 hr at 37°C), (G–I) nocodazole (1 µM for 1 hr at 37°C), or (J–L) calyculin A (200 nM for 20 min at 37°C), and incubated with media containing 2 nM QD (red) for 20 min in the maintained presence of these reagents. Subsequently, these DCs were fixed and stained with FITC-phalloidin to label F-actin (green). Confocal images are consistent with results obtained using real-time two-photon imaging. Scale bar = 10 µm. (3.51 MB TIF) [file pone.0003290.s004.tif]

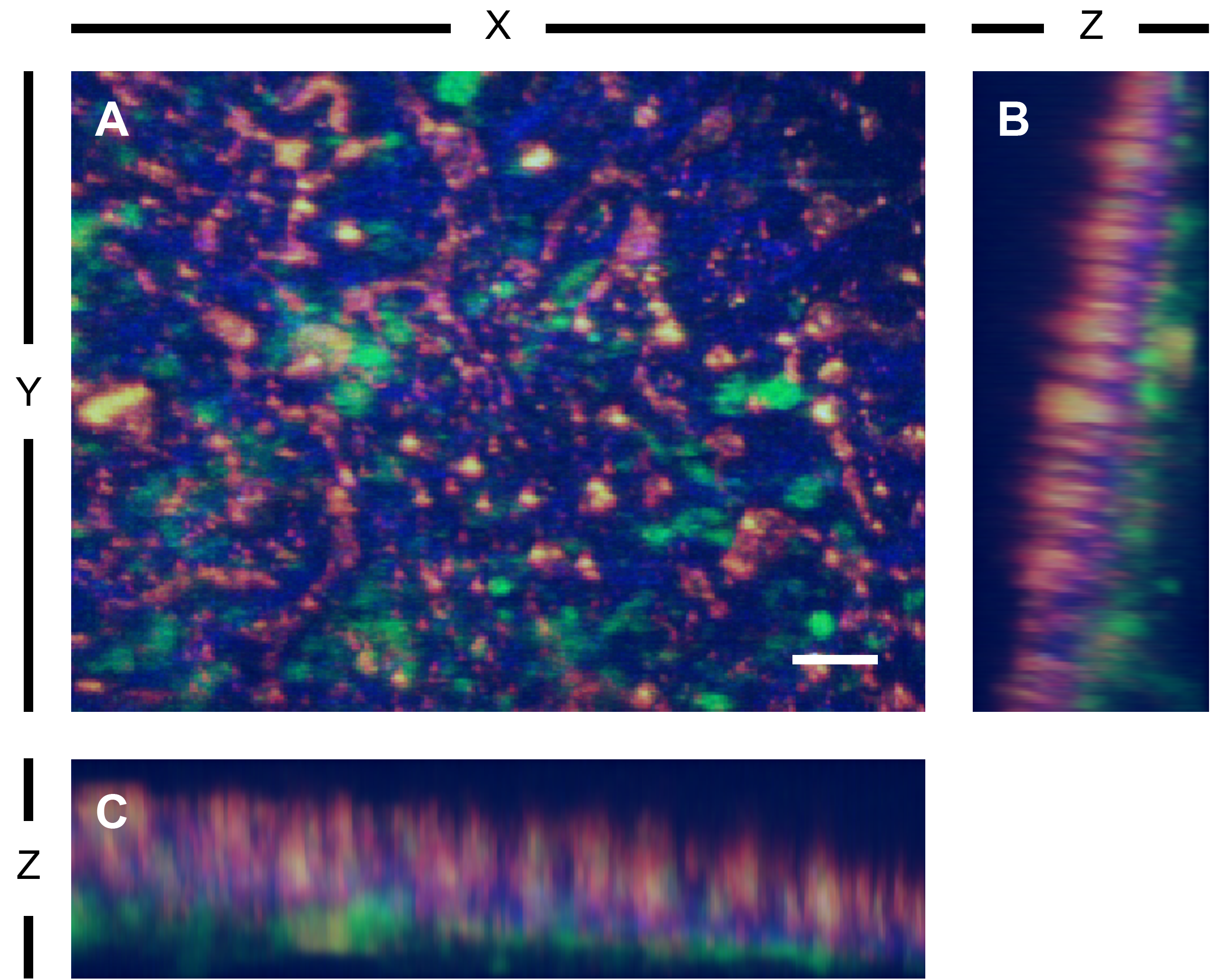

Supplement: Figure S4 — QDs in subcapsular sinus of draining lymph nodes. DCs were labeled in situ by subcutaneously injecting EYFP-CD11c mice with QDs included in 50 µl CFA. Draining lymph nodes were harvested 4 hrs later for imaging. Subcapsular fibers appear blue due to second harmonics. (A) QDs (red) are trapped inside vessels in the capsule and are presumably taken up by subcapsular DCs (green) and macrophages. (B, C) Z sectional views of the capsule show that QDs are not yet present inside the node below the capsule. Scale bar = 20 µm. (5.15 MB TIF) [file pone.0003290.s005.tif]

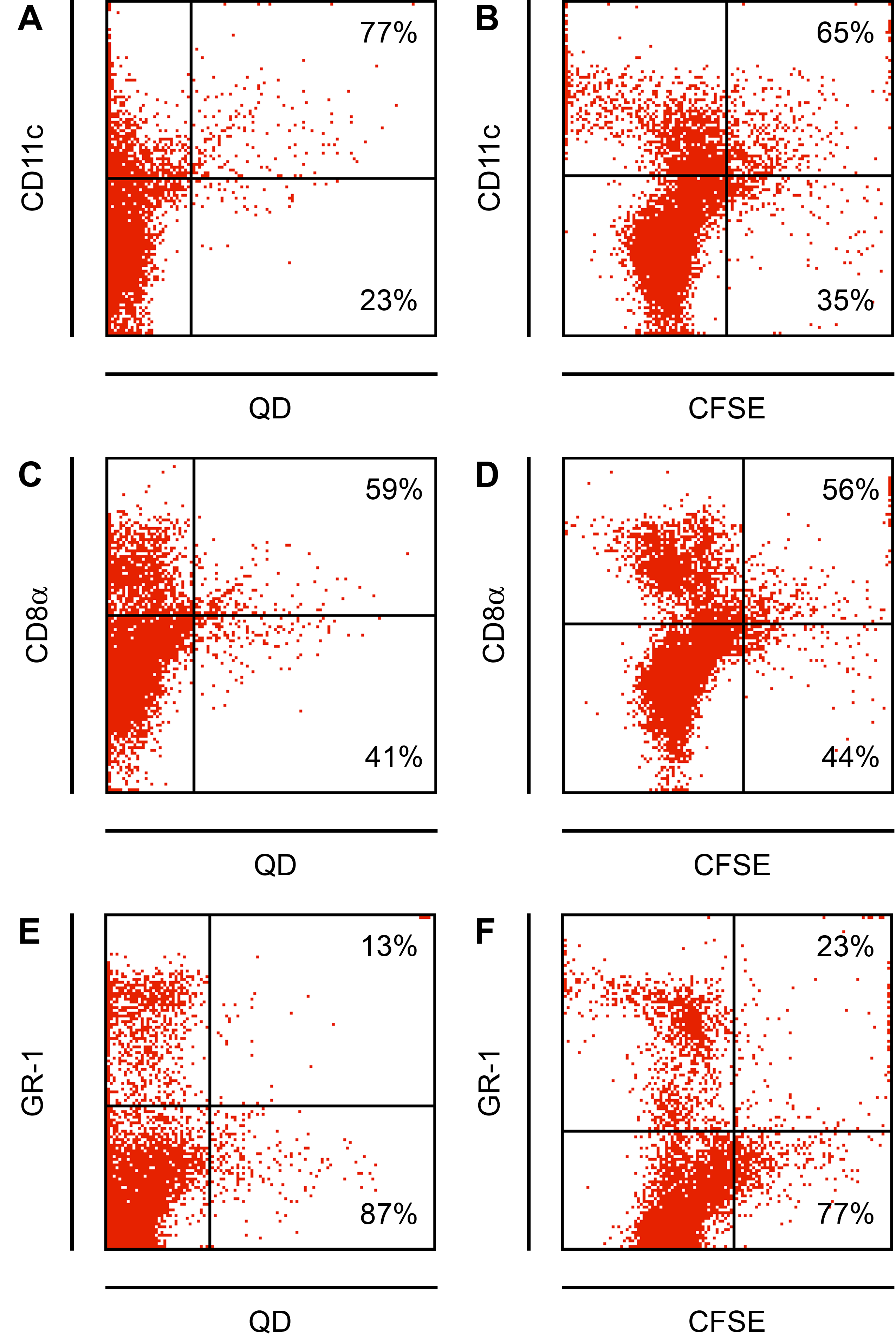

Supplement: Figure S5 — Phenotypic profile of DCs observed by flow cytometry. Phenotypic markers are indicated on the Y-axis of each plot. Distribution of QD+ and CFSE+ cells (percent) for each phenotypic marker is shown on the graph.Note the similarity in distribution of QD+ and CFSE+ cells for each phenotypic marker. (0.86 MB TIF) [file pone.0003290.s006.tif]

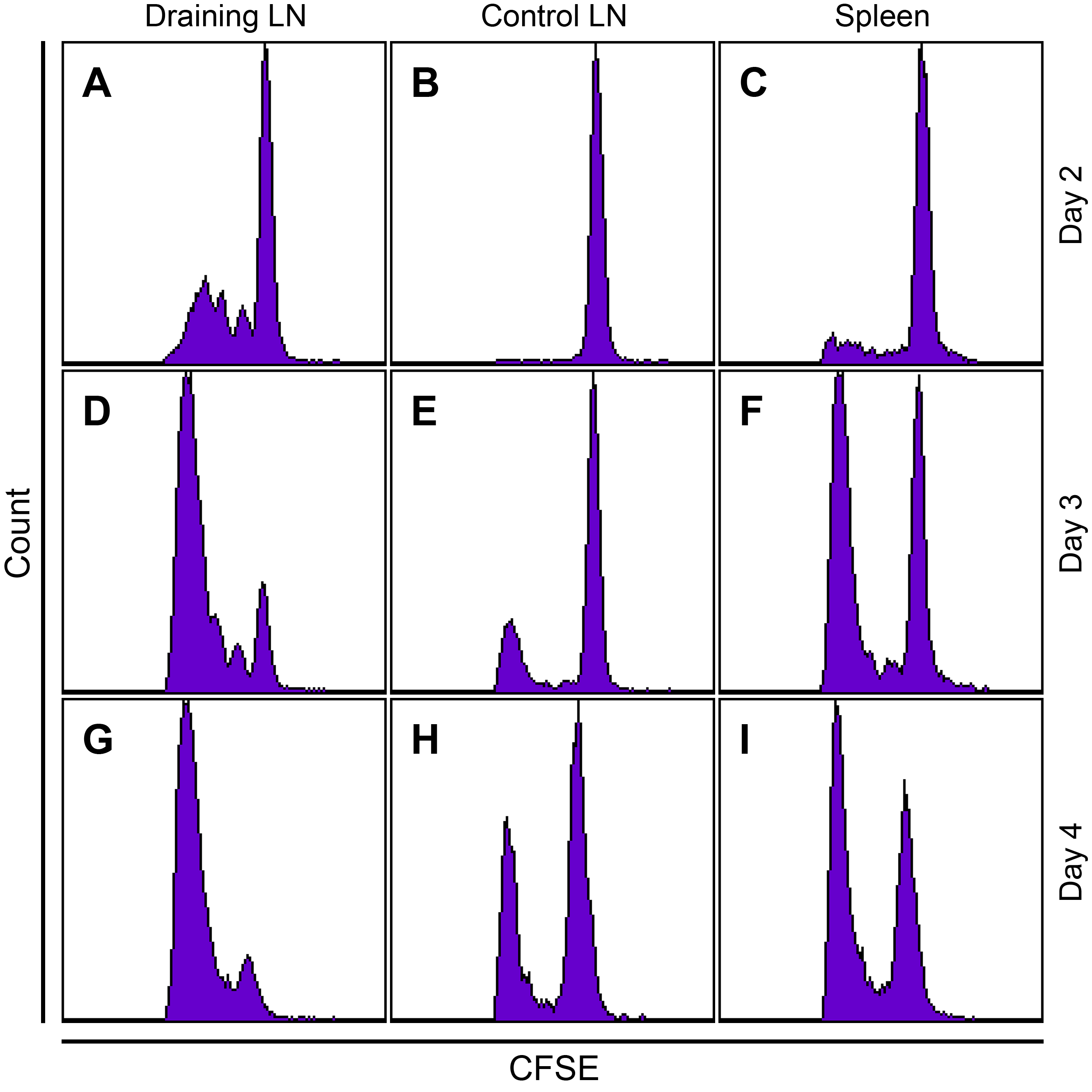

Supplement: Figure S6 — In vivo T cell response at different times following immunization. BALB/c mice were adoptively transferred with ∼4×10^6^ CFSE-labeled DO11.10 T cells, and subsequently immunized with 50 µg of ovalbumin included in 50 µl CFA. Control and draining lymph nodes, and spleens were harvested (A–C) 2 days, (D–F) 3 days, or (G–I) 4 days after injection, and analyzed for T cell activation using flow cytometry. Day 2 (just prior to egress of activated T cells) was chosen as the time point for analysis of T cell activation. (0.32 MB TIF) [file pone.0003290.s007.tif]
